# Realization of a discrete time crystal on 57 qubits of a quantum computer

**DOI:** 10.1126/sciadv.abm7652

**Published:** 2022-03-02

**Authors:** Philipp Frey, Stephan Rachel

**Affiliations:** School of Physics, University of Melbourne, Parkville, VIC 3010, Australia.

## Abstract

Unconventional dynamical phases that violate ergodicity have been a subject of extensive research in recent years. A periodically driven system is naively expected to lose all memory of its initial state due to thermalization, yet this can be avoided in the presence of many-body localization. A discrete time crystal represents a driven system whose local observables spontaneously break time translation symmetry and retain memory of the initial state indefinitely. Here, we report the observation of a discrete time crystal on a chain consisting of 57 superconducting qubits on a state-of-the-art quantum computer. We probe random initial states and compare the cases of vanishing and finite disorder to distinguish many-body localization from prethermal dynamics. We further report results on the dynamical phase transition between the discrete time crystal and a thermal regime, which is observed via critical fluctuations in the system’s subharmonic frequency response and a substantial speedup of spin depolarization.

## Teaser

The time crystal phase has been observed, and a first step toward establishing a dynamical phase diagram has been obtained.

## INTRODUCTION

The phenomenon of spontaneous symmetry breaking is common in nature and characterizes a large class of phases in materials. For instance, the crystal lattice of a solid formed by the nuclei breaks the continuous spatial translation symmetry of the underlying Hamiltonian, as indicated by the mere discrete translation symmetry of the local density operator. In 2012, Wilczek proposed the existence of phases of matter that break continuous time translation symmetry (*1*), dubbed time crystals. Later, their existence in thermal equilibrium was ruled out (*2*). However, time crystals can be stabilized as out-of-equilibrium matter such as periodically driven systems (*3*–*5*), where the dynamical symmetry corresponds to discrete time translations only. Such discrete time crystals (DTCs) (*6*–*10*) exhibit period doubling, tripling, etc., with respect to the periodic driving Hamiltonian. That is, the system returns to its initial state only after some integer multiple of the driving period. Ergodicity predicts that driving heats the system, causing it to thermalize after a certain number of periods. Instead, a DTC is not ergodic and “remembers” its initial state even at late times, in violation of the eigenstate thermalization hypothesis (*11*).

To escape ergodicity, a DTC must be many-body localized (MBL). MBL has been a subject of extensive research in recent years, both theoretical and experimental (*12*–*18*). It can be understood as an emergent integrability through localized integrals of motion. In their presence, the system is prevented from heating to a state that locally resembles thermal equilibrium. For comprehensive reviews on MBL, see (*19*–*23*).

Early and previous experiments have provided important insights into DTCs in a variety of different platforms such as trapped ions (*18*, *24*, *25*), dipolar spin systems (*9*, *26*–*29*), and superfluid quantum gases (*30*–*32*). Most of these experiments fail, however, to satisfy all experimental requirements for realizing DTC spatiotemporal order (*33*): The systems need to be truly many-body, and coherence times must be sufficiently long to be able to distinguish a DTC from short-time transients; the implemented Hamiltonian must contain disordered spin-spin couplings and sufficiently short-ranged interactions. Firmly establishing DTC dynamics requires the ability to prepare arbitrary initial states and perform site-resolved measurements. Present-day quantum computers, so-called noisy intermediate-scale quantum (NISQ) devices, have been suggested as the only platform that currently meets all the requirements above (*33*). Comparatively short coherence times due to noise in the system pose the predominant challenge.

Here, we report the observation of a DTC over a 57-qubit chain on IBM’s quantum computers ibmq_manhattan and ibmq_brooklyn. The best-studied instance is the one-dimensional spin-1/2 chain with disordered nearest-neighbor Ising interactions, driven by an imperfect periodic spin flip (*5*, *24*, *34*).

Prethermal dynamics (*25*, *35*) can mimic the DTC phenomenon for initial states that lie at the edge of the many-body spectrum, whereas true MBL applies to the entire spectrum. Therefore, it is essential to probe random bit strings as initial states. By preparing both fully polarized and random-bit states and varying the simulated disorder, we are able to distinguish between prethermal and the long-sought DTC regime. We further present results on the dynamical phase transition between MBL-DTC and thermal phase.

## RESULTS

### DTC on a quantum computer

We implement a periodically driven Ising chain with quenched disorder and imperfect drive. This is an instance of Floquet evolution, i.e., time evolution defined in terms of a unitary *U* instead of a Hamiltonian *H*. We make use of the ability to directly program any unitary operator acting on a set of qubits, which, due to their nearest-neighbor connectivity, is equivalent to a one-dimensional spin chain. The periodic driving can also be thought of as time evolution under a piecewise-defined Hamiltonian, resulting in discrete time translation symmetry or periodicity. As we will show, the state of this system itself spontaneously breaks this symmetry through period doubling and therefore represents a DTC. The time evolution operator *U* of the Floquet system is defined in terms of two unitaries *U* = *U*_2_*U*_1_: One represents an imperfect global spin flip

$$U_{1}=\text{exp}\;(i\frac{\pi }{2}(1-\epsilon )\sum_{i}X_{i})$$ (1)

where *X_i_* is the Pauli X-gate on the *i*th qubit. The parameter ϵ accounts for a deviation from an ideal spin flip, because qualitatively novel behavior needs to be robust against small perturbations to be considered a true phase of the system. The other unitary corresponds to nearest-neighbor Ising interactions

$$U_{2}=\text{exp}\;(-i\sum_{i}\;J_{i}Z_{i}Z_{i+1})$$ (2)

with the Ising couplings *J_i_* containing the quenched local disorder. The second unitary can be varied by adjusting the Ising interaction couplings {*J_i_*}. We generate disordered sets of couplings by picking each one randomly from an interval *J_i_*ϵ [π/8,3π/8] centered around the mean π/4. While the model defined by the unitaries Eqs. 1 and 2 is integrable and noninteracting, the finite gate errors on the quantum computer introduce effective terms such as longitudinal fields ∼ exp (*i*∑*_i_b_i_Z_i_*); these additional terms make the model nonintegrable and truly many-body (see Discussion).

**Fig. 1.**
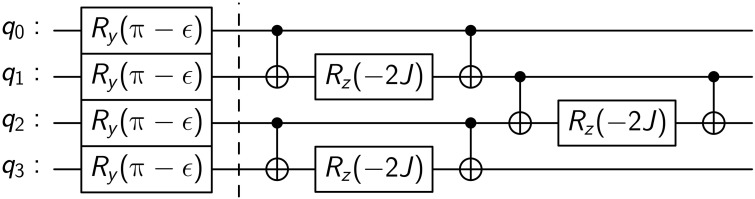
Illustrative four-qubit circuit for one Floquet period. *R_y_* and *R_z_* represent single-qubit rotations around the *y* and *z* axes, respectively. Vertical lines connecting small and large open circles represent CNOT gates. The first part to the left of the vertical dotted line implements the imperfect spin flip, while the latter part to the right of this line implements the nearest-neighbor Ising interactions.

**Fig. 2.**
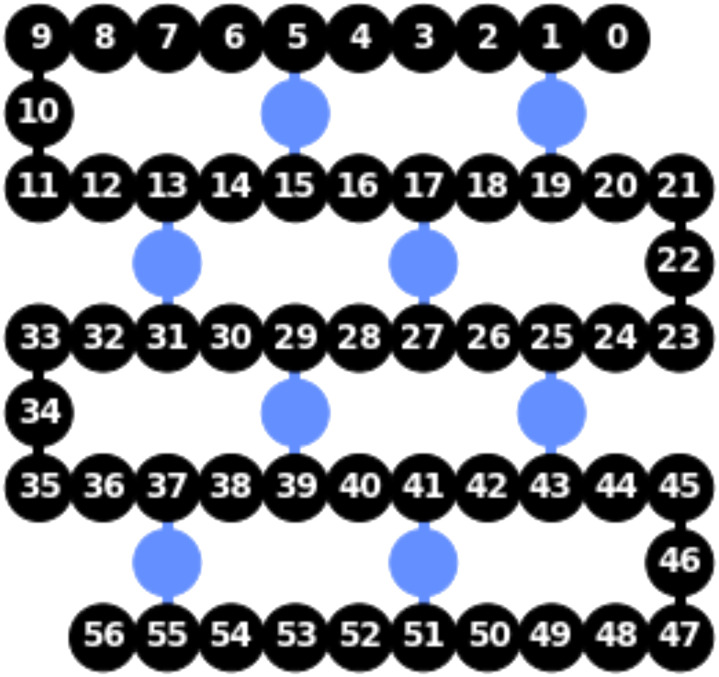
Qubit layout on ibmq_manhattan and ibmq_brooklyn chips with its 65 qubits [for the former, whole-device entanglement was demonstrated recently (*43*)]. The 57 black qubits are used for the simulations of DTC.

A different random bit string as the initial state and a different disorder realization are used for each run of the time evolution. We use a trotterized time evolution, which, for this model, is an exact representation of the unitary *U*. In Fig. 1, we show the circuit decomposition in terms of basis gates for a Trotter step on four qubits. We then measure each qubit in the computational basis corresponding to *Z*. The error rates on current NISQ devices limits the number of Floquet periods to ∼50. By making use of the heavy hex topology of ibmq_brooklyn and ibmq_manhattan (see Fig. 2), we are able to simulate an *N* = 57 site chain and to thereby go far beyond current numerical calculations on any classical computer. These machines have stated average CNOT error rates between 1.1 × 10^−2^ and 3 × 10^−2^ and average readout error rates between 2.5 × 10^−2^ and 3.7 × 10^−2^. The circuit for each time step is run 32,768 times to minimize shot noise.

**Fig. 3.**
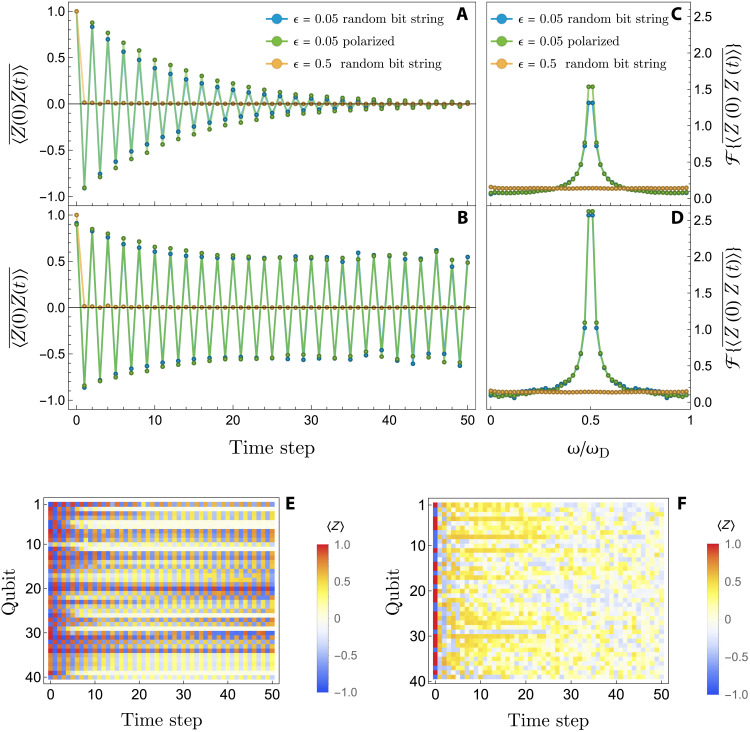
Signatures of DTC and thermal dynamics. (**A**) Averaged spin-spin autocorrelators across time with measurement error mitigation (see Methods) applied to the raw data. (**B**) Same as in (A) but with additional correction of overall decay due to noise. (**C** and **D**) Corresponding frequency spectra. (**E** and **F**) Site-resolved polarizations across time for ϵ = 0.05 (DTC) and ϵ = 0.05 (thermal phase), respectively. Of the total 57 qubits, we show those 40 qubits that operate within reasonable bounds on error rates, as judged by our mitigation algorithm (see Methods).

The realization of the DTC can be observed by site-resolved measurement of the spin-spin autocorrelators across time, 〈*Z_i_*(0)*Z_i_*(*t*)〉. It is convenient to consider the averaged autocorrelator $\bar{<Z_{i}(0)Z_{i}(t)>}=\sum_{i}\langle Z_{i}(0)Z_{i}(t)\rangle /N_{q}$, where *N_q_* is the number of qubits. Rapid decay of these correlators is expected for the thermal regime; in contrast, the oscillations in theory persist indefinitely for the DTC because of MBL. The inherent noise of NISQ devices causes depolarization of the qubits and represents the main challenge for distinguishing between DTC and thermal regime. Even in the ideal case of ϵ = 0, where *U* should not generate any entanglement for an initial product state, we observe a finite rate of decay. Figure 3A shows the averaged autocorrelator after correcting for measurement errors. For ϵ = 0.05, we observe persistent oscillations as a hallmark of the realized DTC. Deviations from ±1 are attributed to a combination of noise and the fact that the conserved operators of the effective MBL Hamiltonian do not exactly coincide with the set of operators {*Z_i_*}; hence, the *Z_i_* are only partly conserved at late times. Nonetheless, the oscillators are stable over 50 Floquet periods and are clearly distinct from prethermal dynamics (*25*, *33*). Even with finite coherence time, one can still clearly distinguish between the rapid decay of a thermal system for ϵ = 0.5 and the DTC for ϵ = 0.05. In addition, we show the latter for a fully polarized initial state with almost identical results. Using reference data obtained for ϵ = 0 allows us to rescale the autocorrelations because, in this case, any deviation from a perfect oscillation between −1 and 1 is caused by noise. The precise error mitigation scheme is more involved and has been detailed in Methods. In Fig. 3B, we show the fully error-mitigated data, corresponding to the data shown in Fig. 3A, including the correction for noise-induced depolarization. The data for ϵ = 0.5 lead to immediate thermalization; for ϵ = 0.05, we observe the persistent oscillations of a DTC, irrespective of which initial state we choose (see the Supplementary Materials). We note that the error mitigation scheme does not boost the observed signal beyond factoring out the baseline decay rate, as described in Methods.

The DTC phase can also be observed through a pronounced peak in the Fourier spectrum of <*Z_i_*(0)*Z_i_*(*t*)> at half the driving frequency ω_D_ = 2π*T*^−1^ for each spin. We show the qubit-averaged Fourier spectrum in Fig. 3C (D) that corresponds to the measurement error–mitigated (fully error-mitigated) data. Even in the absence of any error mitigation, the peak at ω_D_/2 is pronounced; the mitigation scheme almost doubles the peak height.

In Fig. 3 (E and F), we show site-resolved measurements for representative data points within the DTC phase and the thermal phase, respectively, for different initial states. Displayed are the 40 qubits that fulfill the criteria for sufficiently low error rates as defined in Methods. The former displays, to varying degree, staggered spin polarizations across time for each individual qubit, while the latter shows rapid depolarization across the entire system. This shows a clear distinction between two different dynamical phases. One phase is characterized by the breaking of ergodicity through MBL and, furthermore, the spontaneous breaking of an emergent Ising symmetry, resulting in period doubling and therefore time crystalline dynamics. The other phase exhibits standard ergodicity and thus rapidly evolves toward a thermal state.

### Dynamical phase transition

In the following, we focus on the dynamical phase transition from the DTC regime to the thermal phase. It can be shown (*5*) that time evolution under the above Floquet unitary over an even integer number of periods is unitarily equivalent to time evolution with an effective Hamiltonian

$$VU(2\mathrm{nT})\;V^{†}\approx e^{-i2{\mathrm{nH}}_{\text{TFIM}}T}$$

The approximate sign indicates that the representation in terms of a conserved Hamiltonian is not correct out to temporal infinity and that the effective Hamiltonian itself contains higher-order terms that we neglect (*36*). *V* is a finite depth unitary operator that depends on the particular disorder, and the random transverse field Ising model (TFIM) Hamiltonian is given by

$$H_{\text{TFIM}}=\sum_{i}\;{\tilde{J}}_{i}Z_{i}Z_{i+1}+{\tilde{B}}_{i}^{x}\;X_{i}$$

The parameters ${\tilde{J}}_{i}^{z}$ and ${\tilde{B}}_{i}^{x}$ are disordered, and varying ϵ essentially translates into varying the mean $\bar{{\tilde{B}}_{i}^{x}}$. The random TFIM exhibits an Ising symmetry that its eigenstates either share or spontaneously break, depending on the magnitude of $\bar{{\tilde{B}}_{i}^{x}}$. The latter case corresponds to the DTC phase of the Floquet system.

**Fig. 4.**
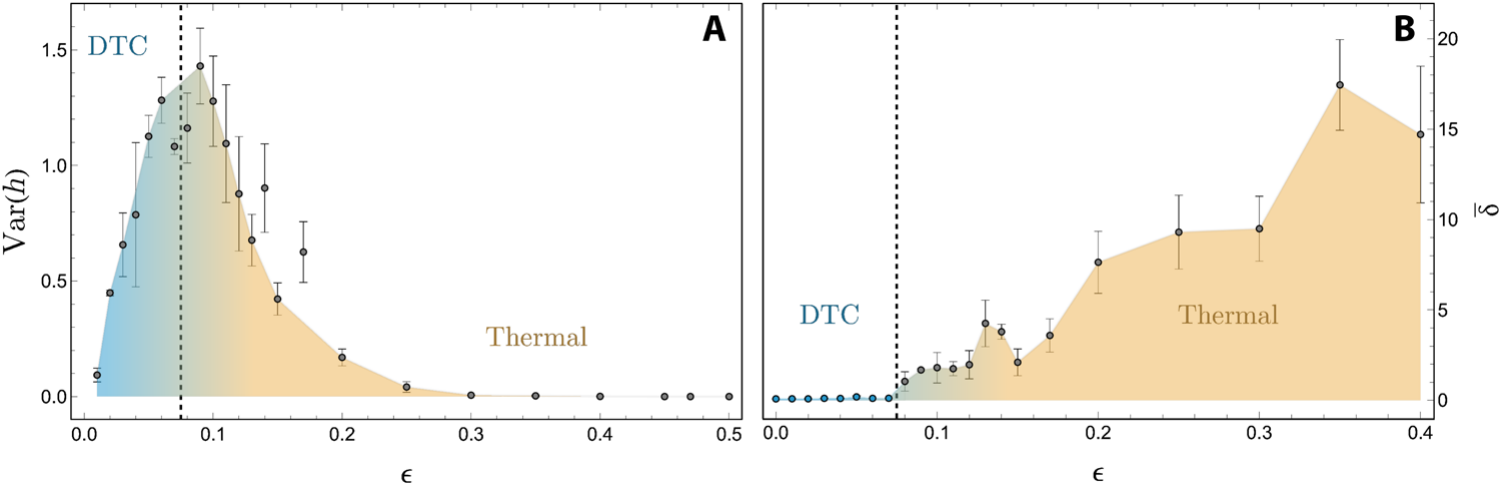
Dynamical phase transition. (**A**) Critical fluctuations in the subharmonic frequency response. Filling and dotted lines are a guide to the eye. (**B**) Average decay constant $\bar{\delta }$ across qubits. The transition from DTC to the thermal phase is indicated by the rather sudden increase in $\bar{\delta }$. The dashed vertical lines in (A) and (B) indicate the dynamical phase transition at ϵ_c_ ≈ 0.075.

This leads us to the first indicator of the phase transition, namely, critical fluctuations at the transition between the DTC and the thermal phase in the order parameter that spontaneously breaks the Ising symmetry in one phase but not in the other. The order parameter of the random Ising transition is the *z* magnetization, and a finite value will result in stable oscillations at ω = ω_D_/2 due to the periodic flip operation. Defining *h_i_* = ∣ℱ{〈*Z_i_*(*t*)〉}(ω_D_/2)∣, with ℱ{ · } representing the Fourier transform, one can conclude that the variance across the chain Var({*h_i_*}) should vanish in the DTC phase and in the thermal phase. At the transition, we instead expect critical fluctuations to produce a finite value. For short chains (*N* ∼ 10), one has to average over several disorder realizations to obtain a clear signal because the behavior at the transition is sensitive to the particular choice of disorder. Our system of *N* = 57 sites seems to produce a rather clear signal without this averaging process, as one might expect. Figure 4A shows the variance as a function of ϵ after we have reduced the noise by applying error mitigation (see Methods). A pronounced peak indicates the phase transition and allows us to extract ϵ_c_ ≈ 0.075 (the peak’s maximum) as an estimate for the phase transition. Finite values at very small values of ϵ may be attributed to the aforementioned fact that only part of *Z_i_* is actually conserved at late times.

The second indicator of the transition is the average decay constant $\bar{\delta }$ of the local polarization across the chain. With every spin roughly following an exponential decay (barring initial transients), i.e., ∣ < *Z_i_* > ∣ (*t*) ∝ exp ( − δ*_i_t*), we define $\bar{\delta }=\sum_{i}\delta_{i}/N_{q}$. While finite even in the ideal case of ϵ = 0 due to noise, $\bar{\delta }$ is expected to increase substantially when transitioning into the thermal phase. The transition found in Fig. 4B nicely agrees with ϵ_c_ ≈ 0.075.

## DISCUSSION

The model as defined in Eqs. 1 and 2 is noninteracting. However, using process tomography, we were able to analyze the systematic error contributions to the effective Hamiltonian associated with the Ising interaction. The dominant terms generated correspond to longitudinal fields and effectively contribute a third unitary *U*_3_ = exp {(−*i*∑*_i_b_i_Z_i_*)}, where the random amplitudes *b_i_* can be at least as large as π/25. In addition, further spin-spin interactions are generated (see the Supplementary Materials for details). We therefore argue that the actual model, as implemented on the quantum computer, is truly interacting and thus qualifies as MBL. This is in line with the reasoning and numerical evidence put forward in (*33*). As we do not explicitly add an additional longitudinal field unitary *U*_3_ by hand, our circuit depths are significantly reduced. This allows us to minimize the noise in our signal while taking advantage of the ubiquitous gate errors to meet one of the requirements for MBL.

It is well known that special states at the edge of the spectrum, such as a fully polarized chain or a Neel state, can exhibit prethermal dynamics that resembles DTC even if the full spectrum is not MBL. In that case, one expects a strong dependence of the observed dynamics on the initial state. Random bit strings would show a much faster rate of depolarization than any of these special states. By comparing the data obtained for a fully polarized chain with the generic random bit string (see Fig. 3), we conclude that there seems to be no such strong dependence, especially when noise is taken into account. In the Supplementary Materials, we also show a fully polarized chain as well as a random bit string as initial states without any disorder in the Ising couplings in the driving Hamiltonian, leading to a notable reduction in the oscillation amplitude as expected.

The DTC phase and the transition to the thermal phase could be observed for a substantially larger system size than in previous experiments, and we were able to make a first step toward establishing a dynamical phase diagram for this system. With increasing fidelity of NISQ devices in the near-term future, we can expect to shine light on some of the open problems in MBL, such as finite-size scaling and the transition between the thermal phase and the MBL paramagnet.

Note added: After being informed about (*33*), we have updated the manuscript accordingly. During completion of this work, we became aware of two related works. In (*37*), a DTC phase using an array of eight capacitively coupled transmon qubits is demonstrated experimentally. In (*38*), the observation of a DTC on a quantum computer with 20 qubits was reported. Our main results in Fig. 3 and Fig. S1 are in agreement with (*38*) but were derived independently.

## METHODS

### Error mitigation

For current NISQ devices, error mitigation is crucial. Notably, there has been tremendous progress in developing error mitigation schemes and demonstrating stability of quantum circuits against quantum gate errors and shot noise (*39*–*42*). For this work, we have developed a scheme based on our understanding of the dominant contributions to the total error. Our error mitigation scheme is tailored to deal with measurement errors and the depolarization due to the environment.

### Measurement error

The first major contribution is the error associated with measurement of the individual qubit’s state. For a small set of *n* qubits, one can calibrate and correct for this error by initializing the register in each of the 2*^n^* possible computational basis states and measuring <*Z*> on every qubit. However, for a large register, this is not feasible, and so, we assume that the measurement errors on different qubits are approximately uncorrelated. Because the measurement errors are not consistent across different runs over the course of hours or days, one would have to recalibrate before every experiment and assume that the drift is minimal in between calibration and the actual simulation. Systematic errors that offset the polarization might also be introduced by gate errors resulting from the application of trotterized time evolution. To tackle both of these effects, we use an empirical approach. Along with every simulation run for a given finite ϵ and fixed disorder and initial state, we also perform a reference simulation with ϵ = 0 and otherwise unchanged conditions. Any bit string is an eigenstate for the unperturbed unitary. This allows us to conclude that any deviation from a constant local polarization is due to noise and errors. The combination of measurement errors and systematic gate errors at late times is characterized by a pair of effective error parameters η_0_ and η_1_ for each qubit. η_0_ denotes the effective probability of erroneously measuring a 1 given that the qubit is in state 0, and η_1_ denotes the effective probability of obtaining a 0 from a 1 state. We are interested in the expectation value <*Z*>, and with $\bar{\eta }≔(\eta_{0}+\eta_{1})/2$ and Δ ≔ η_0_ − η_1_, one can easily show that the corrected expectation value <*Z*>_corr_ can be obtained from the measured one <*Z*>_meas_ via

$${\langle Z\rangle }_{\text{corr}}=\frac{{\langle Z\rangle }_{\text{meas}}+\Delta }{1-2\bar{\eta }}$$ (3)

This empirical approach requires the input of at least two data points at different times. To not overcorrect the measurements at early times, where systematic gate errors have not yet accumulated to the same extent as for late times, we choose to normalize the polarizations to ∣< *Z* > (*t* = 0)∣ = 1

$${\langle Z\rangle }_{\text{corr}}=\frac{{\langle Z\rangle }_{\text{meas}}-{\langle Z\rangle }_{\text{final}}}{|{\langle Z\rangle }_{\text{meas}}(t=0)-{\langle Z\rangle }_{\text{final}}|}$$ (4)

<*Z*>_final_ represents an average over a few data points at the latest simulated times. The average is taken to avoid the effects of changing conditions over the course of the simulation. From this, one can, in principle, extract the effective empirical error parameters

$$\eta_{0}=(1-|{\langle Z\rangle }_{\text{meas}}(t=0)-{\langle Z\rangle }_{\text{final}}|-{\langle Z\rangle }_{\text{final}})/2$$ (5)

$$\eta_{1}=(1-|{\langle Z\rangle }_{\text{meas}}(t=0)-{\langle Z\rangle }_{\text{final}}|+{\langle Z\rangle }_{\text{final}})/2$$ (6)

### Depolarization due to environment

In addition to the measurement and gate errors, one can also observe an overall exponential decay of polarization due to the qubits thermalizing with their environment. To compensate for this decay, we would like to rescale the data accordingly. We use the following algorithm to distinguish between a qubit being nonpolarized at late times due to internal thermalization versus due to erroneous spin flips:

1. Introduce a cutoff parameter *W*_0_ ∈ [0,1], where 1 corresponds to the maximal polarization of a qubit. Qubits whose average polarization over 5 time steps drops below *W*_0_ after the initial 13 time steps for ϵ = 0 are excluded from the evaluation (further justification below).

2. Introduce a fixed threshold *W*_f_ < *W*_0_ for finite values of ϵ. Qubits that lie within the interval [ −*W*_f_, *W*_f_] after the initial time evolution are assumed to approach vanishing magnetization due to thermalization, in which case, the accumulated gate error in the magnetization is less relevant (<σ*^z^* > = 0 is a fixed point as far as the Trotter gate errors are concerned). Therefore, we do not rescale the subsequent data points for these qubits.

3. For qubits outside of this interval, one can assume that most of the damping is due to the same depolarizing noise that was observed previously for ϵ = 0. We rescale the subsequent data points with an exponential fit obtained from the ϵ = 0 data for this qubit but adjusted by the ratio of average polarization for a given finite ϵ to the corresponding average polarization for vanishing ϵ. With $m_{i}^{\left(\epsilon \right)}(t)$ denoting the magnetization of the *i*th qubit at time *t* as measured on the quantum computer and corrected for measurement errors, the exponential fit of the reference data at ϵ = 0 takes the form

$$\frac{1}{2}[m_{i}^{\left(0\right)}(t)+\text{sign}(m_{i}^{\left(0\right)}(t))]=a_{i}^{\left(0\right)}e^{-b_{i}^{\left(0\right)}t}+c_{i}^{\left(0\right)}$$ (7)

with fit parameters $a_{i}^{\left(0\right)}$, $b_{i}^{\left(0\right)}$, and $c_{i}^{\left(0\right)}$. We define the rescaled magnetization $M_{i}^{\left(B\right)}(t)$ via

$$M_{i}^{\left(\epsilon \right)}(t)=\frac{\bar{m_{i}^{\left(\epsilon \right)}}}{\bar{m_{i}^{\left(0\right)}}}\frac{m_{i}^{\left(\epsilon \right)}(t)+\text{sign}(m_{i}^{\left(\epsilon \right)}(t))}{a_{i}^{\left(0\right)}e^{-b_{i}^{\left(0\right)}t}+c_{i}^{\left(0\right)}}-\text{sign}(m_{i}^{\left(\text{on}\right)}(t))$$ (8)

The bar denotes an average over 5 time steps after the initial 13 time steps. Omitting the first few iterations serves to avoid the influence of initial transients on the observed signature. On the basis of numerical simulations for smaller chains, one expects the individual spins to approach an almost steady state after initial, short-lived transients. One therefore predicts that most spins would exhibit an exponential decay even for finite ϵ. Because not all the 57 qubits seem to operate within the margins given by the stated error rates, we additionally remove those few ones that deviate substantially from the exponential decay model as judged by convergence of our fit. The above procedure requires us to omit qubits whose error rates are sufficiently high to move their absolute value polarization into the interval [0, *W*_0_] even for ϵ = 0, because we would otherwise confuse them for ones that are thermal. This justifies step 1 in the above error mitigation scheme.

The same error mitigation algorithm was applied to all the data in Fig. 3B. Rescaling of the late-time data points only occurs if every one of the abovementioned criteria is met. This ensures that instances of markedly dampened time evolution, as displayed by the yellow curve in Fig. 3, remain dominated by the increased rate of depolarization.

To ensure that the arbitrary threshold *W*_0_ does not crucially affect the results, we process the data using a range of different values. The peak associated with critical fluctuations and the signature of increased damping remain quite stable across a wide range. The same applies to the choice of *W*_f_ that enters in the definition of the interval. Here, values very close to 1 produce an algorithm that is too sensitive to fluctuations in the error rates, while values close to 0 suppress the signal. The results presented in this paper were evaluated using *W*_0_ = 0.15 and *W*_f_/*W*_0_ = 2/3. We find that typically ∼40 to 45 qubits enter the evaluation.

## Supplementary Materials

### This PDF file includes:

Sections S1 to S4 Figs. S1 to S3 Table S1 References
