# Supplementary material for: Realization of a discrete time crystal on 57 qubits of a quantum computer
Source: Sci Adv. 2022 Mar 2;8(9):eabm7652. doi: 10.1126/sciadv.abm7652 (PMC8890700; doi:10.1126/sciadv.abm7652)
Supplement: Supplementary file 1 — Sections S1 to S4 Figs. S1 to S3 Table S1 References [file sciadv.abm7652_sm.pdf]

Science Advances
AAAS

Supplementary Materials for

Realization of a discrete time crystal on 57 qubits of a quantum computer

Philipp Frey and Stephan Rachel*

*Corresponding author. Email: stephan.rachel@unimelb.edu.au

Published 2 March 2022, *Sci. Adv.* **8**, eabm7652 (2022)
DOI: 10.1126/sciadv.abm7652

This PDF file includes:

Sections S1 to S4
Figs. S1 to S3
Table S1
References

S1. COMPARISON OF DIFFERENT INITIAL STATES AND ABSENCE OF DISORDER

Here we show the results for a Neel state, in addition to Fig. 2 in the main text. Moreover, we study both a fully polarized and a random-bit state for vanishing Ising-even disorder in the driving Hamiltonian. The decay of the Neel state matches that of a polarized or random initial state very closely in the presence of disorder, indicating MBL. Without disorder the random initial state depolarizes significantly faster than any of these. This becomes even more apparent after applying the error mitigation where the inconsistent oscillations at late times typically indicate that the unmitigated signal is too weak and the result of the mitigation scheme become error dominated. A fully polarized chain without disorder in the driving Hamiltonian seems to decay signifantly faster compared to the disordered system as well. The difference becomes more apparent after the error mitigation. However, it seems to be more stable than the random bit string, indicating that special initial states can exhibit prethermal dynamics that mimics DTC even in the absence of MBL.

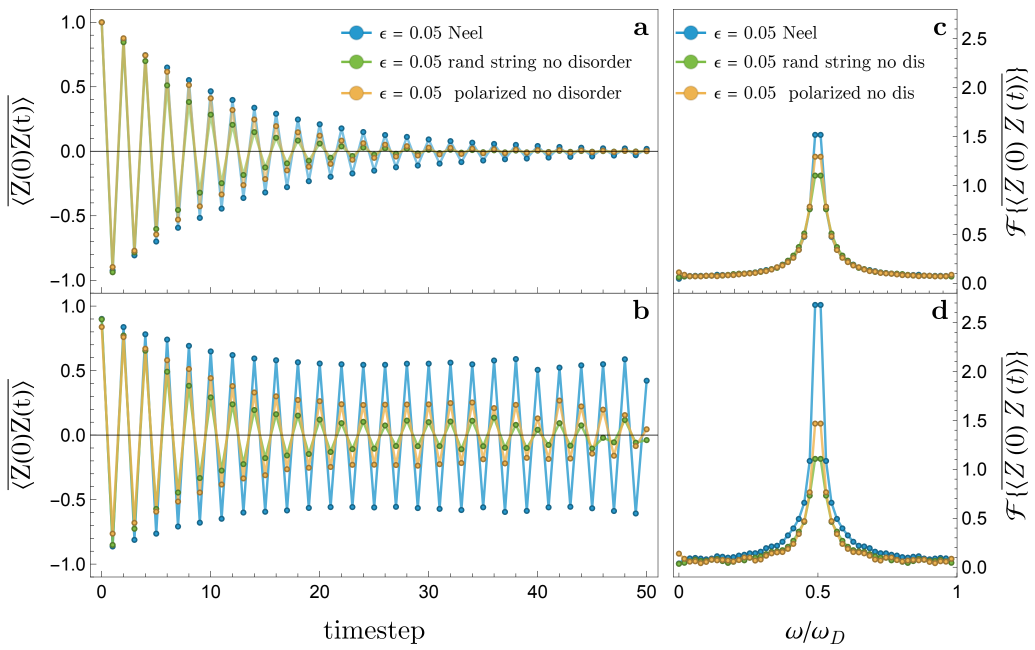

FIG. S1. **DTC stability of special states and translation invariant chains.** **a** Averaged spin-spin auto-correlators across time with measurement error mitigation (see Methods) applied to the raw data. **b** same but with additional correction of overall decay due to noise. **c** and **d** show corresponding frequency spectra.

S2. LOCAL SPIN OBSERVABLES

Here we show the site resolved Fourier spectrum (absolute value) of oscillations in $\langle Z(t)\rangle$ for different values of ϵ. This equivalent to looking at $\langle Z(0)Z(t)\rangle$ since the difference in sign does not affect the absolute values of the individual Fourier spectra. The pronounced peak at half the driving frequency ω_D at low ϵ indicate the DTC phase. Fig.S2 a with $\epsilon = 0.02$ is supposedly well inside the DTC phase but clearly shows that there is a finite variance in the amplitude of the subharmonic frequency response. This is due to the fact that only a finite fraction of each spins Z-component is conserved and this fraction varies accross the chain due to the quenched disorder. Well within the thermal phase none of the spins are oscillating at late times and hence the frequency response is almost completely flat, see Fig.S2 c. Thus the associated variance vanishes as well. In the vicinity of the phase transition the excess variance compared to the case of small ϵ becomes very apparent in the site resolved spectrum, Fig.S2 b.

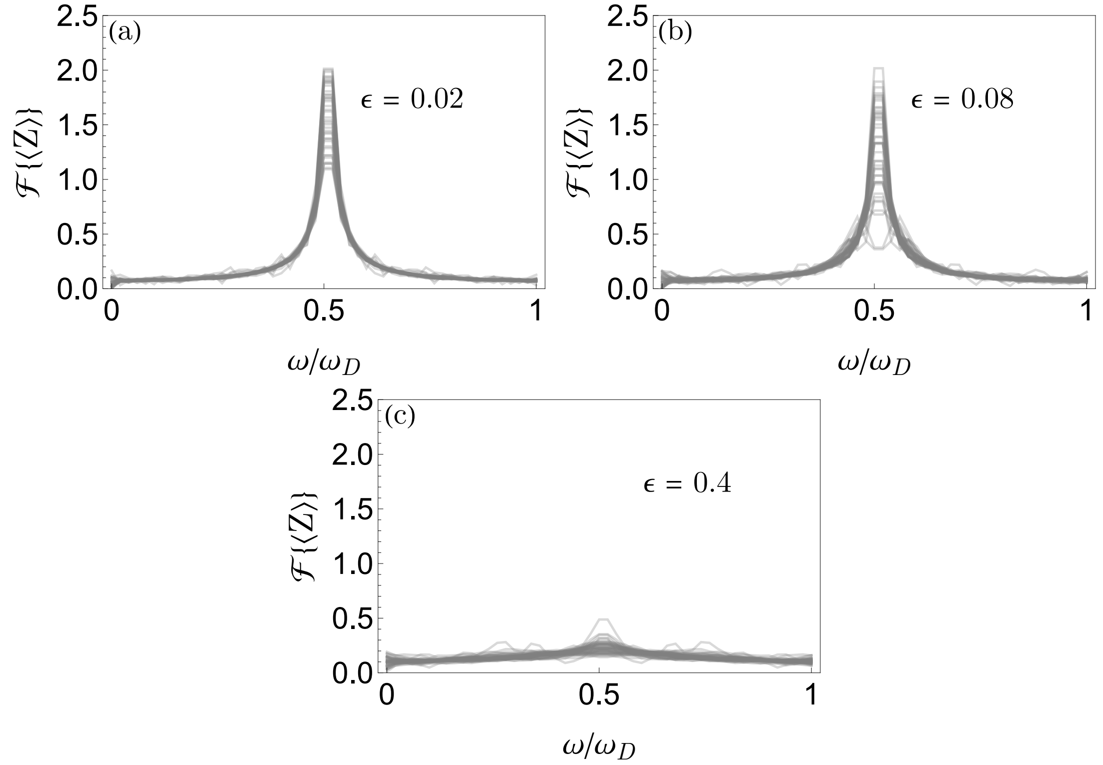

FIG. S2. **Site resolved spin observables a** Overlay of Fourier spectra of each qubit for $\epsilon = 0.02$. **b** and **c** show the same for $\epsilon = 0.08$ and $\epsilon = 0.4$ respectively.

S3. SITE RESOLVED DTC ACROSS ENTIRE CHAIN

Here we show the site resolved oscillations after applying full error mitigation but without removing any qubits based on the filter criteria presented in the Methods section. It is clear that the increasing signal towards late times on some qubits (here qubits 20, 21, 22, 24, 30) is an artefact of the mitigation scheme and typically avoided when filtering out qubits based on particularly high error rates. It also seems that many of the qubits that thermalize rapidly and completely (here qubits 11, 25, 27, 42, 45) are typically removed by our scheme, indicating that this is due to significantly higher error rates.

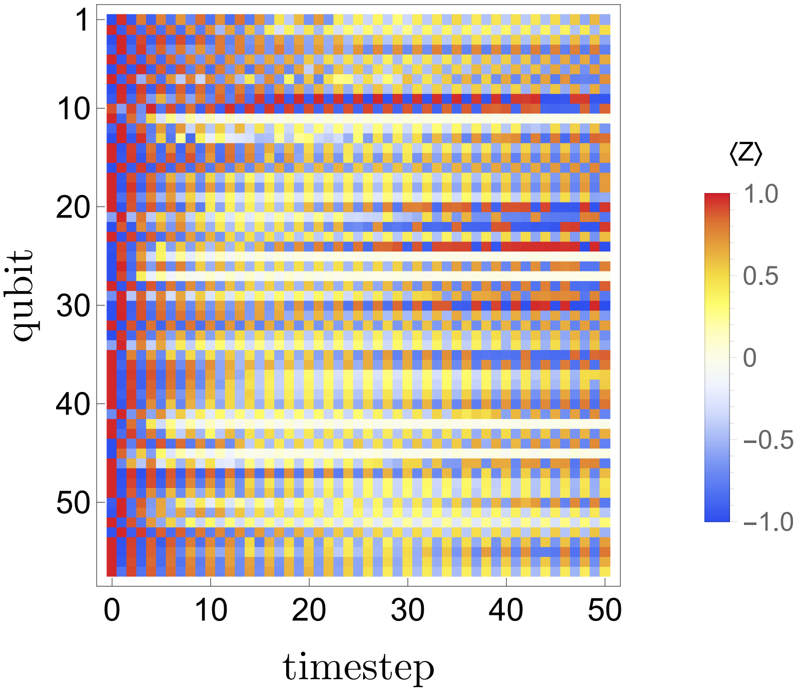

FIG. S3. **Site resolved DTC including all qubits.** Fully error mitigated data obtained for $\epsilon = 0.05$ on *imb_brooklyn*.

S4. PROCESS TOMOGRAPHY

In the following we present the results for process tomography performed on a single Floquet time step on three qubits. Process tomography consists of preparing the quantum register in a variety of different initial states, executing the quantum circuit in question, and then measuring the output state with respect to different bases. From the shot statistics one extracts an estimate of the quantum map associated with this process, which may differ from the ideal unitary due to error contributions.

In particular one can define the post-gate error generator for the map, $L = \log\big(G * H^{-1}\big)$, where G is the process matrix for the estimate and H is the process matrix for the ideal gate. This describes all Markovian errors as if they occurred after the gate. The error generator decomposes like a Lindbladian into coherent Hamiltonian terms and dissipative terms. The Hamiltonian generators are the relevant part for us, since they constitute additional effective terms in the Floquet unitary that are systematic and not included in the original model. Since the data for each time step in our simulation is averaged over many shots, it is affected by exactly these systematic terms.

Table S1 shows the coefficients of the dominant Pauli terms in the effective "post-gate Hamiltonian" H_{add}, i.e., our unitary U gets modified to $\exp(-i\, H_{\text{add}}) * U$. In particular, we observe the local and random Z rotations, mentioned in the main text, as well as significant weight-two interactions (i.e., terms such as $Z_i X_j$), thus substantiating our claim that the effective model is interacting. To be specific, H_{add} contains all the terms listed in Table S1, i.e., $H_{\text{add}} = 0.118 X_3 + 0.085 Y_3 + 0.126 Z_3 + 0.023 X_2 + \ldots$, where we dropped the remaining nine terms.

Pauli operator	coefficient
IIX	0.118
IIY	0.085
IIZ	0.126
IXI	0.023
IYI	0.012
IZI	0.033
IZX	0.038
IZY	0.033
XII	0.024
IXI	0.023
YZI	0.023
ZII	0.037
ZYI	0.017

TABLE S1. **Dominant Pauli operators contributing to the effective additional unitary.** Terms and coefficients were derived via process tomography on three qubits. For instance, "IIX" is a short notation for the term $I_1 I_2 X_3$. Besides non-trivial spin-spin interactions, longitudinal fields (IIZ, IZI, ZII) are present with amplitudes 0.126, 0.033 and 0.037, respectively.

We emphasize that extracting the Pauli operators and their amplitudes from the quantum computer data is performed on a classical computer, preventing us from extracting the same information for all 57 qubits. This is, however, not necessary: the results of our process tomography on three qubits are expected and appear to be consistent with previous analysis of IBM's qubits [44, 45]. Our findings outlined in Table S1 are thus representative for the 57 qubit circuits in the main text.

REFERENCES AND NOTES

1. F. Wilczek, Quantum time crystals. *Phys. Rev. Lett.* **109**, 160401 (2012).

2. H. Watanabe M. Oshikawa, Absence of quantum time crystals. *Phys. Rev. Lett.* **114**, 251603 (2015).

3. V. Khemani, R. Moessner S. L. Sondhi, A brief history of time crystals (2019); arXiv:1910.10745.

4. K. Sacha, J. Zakrzewski, Time crystals: A review. *Rep. Prog. Phys.* **81**, 016401 (2017).

5. N. Y. Yao, A. C. Potter, I.-D. Potirniche A. Vishwanath, Discrete time crystals: Rigidity, criticality, and realizations. *Phys. Rev. Lett.* **118**, 030401 (2017).

6. V. Khemani, A. Lazarides, R. Moessner S. L. Sondhi, Phase structure of driven quantum systems. *Phys. Rev. Lett.* **116**, 250401 (2016).

7. D. V. Else, B. Bauer C. Nayak, Floquet time crystals. *Phys. Rev. Lett.* **117**, 090402 (2016).

8. R. Moessner, S. L. Sondhi, Equilibration and order in quantum floquet matter. *Nat. Phys.* **13**, 424–428 (2017).

9. S. Choi, J. Choi, R. Landig, G. Kucsko, H. Zhou, J. Isoya, F. Jelezko, S. Onoda, H. Sumiya, V. Khemani, C. von Keyserlingk, N. Y. Yao, E. Demler M. D. Lukin, Observation of discrete time-crystalline order in a disordered dipolar many-body system. *Nature* **543**, 221–225 (2017).

10. D. V. Else, C. Monroe, C. Nayak N. Y. Yao, Discrete time crystals. *Annu. Rev. Condens. Matter Phys.* **11**, 467–499 (2020).

11. M. Srednicki, Chaos and quantum thermalization. *Phys. Rev. E* **50**, 888–901 (1994).

12. V. Oganesyan, D. A. Huse, Localization of interacting fermions at high temperature. *Phys. Rev. B* **75**, 155111 (2007).

13. D. M. Basko, I. L. Aleiner B. L. Altshuler, Possible experimental manifestations of the many-body localization. *Phys. Rev. B* **76**, 052203 (2007).

14. A. Pal, D. A. Huse, Many-body localization phase transition. *Phys. Rev. B* **82**, 174411 (2010).

15. I. V. Gornyi, A. D. Mirlin D. G. Polyakov, Interacting electrons in disordered wires: Anderson localization and low-*T* transport. *Phys. Rev. Lett.* **95**, 206603 (2005).

16. E. V. H. Doggen, F. Schindler, K. S. Tikhonov, A. D. Mirlin, T. Neupert, D. G. Polyakov I. V. Gornyi, Many-body localization and delocalization in large quantum chains. *Phys. Rev. B* **98**, 174202 (2018).

17. D. A. Huse, R. Nandkishore, V. Oganesyan, A. Pal S. L. Sondhi, Localization-protected quantum order. *Phys. Rev. B* **88**, 014206 (2013).

18. J. Smith, A. Lee, P. Richerme, B. Neyenhuis, P. W. Hess, P. Hauke, M. Heyl, D. A. Huse C. Monroe, Many-body localization in a quantum simulator with programmable random disorder. *Nat. Phys.* **12**, 907–911 (2016).

19. R. Nandkishore, D. A. Huse, Many-body localization and thermalization in quantum statistical mechanics. *Annu. Rev. Cond.ens Matter Phys.* **6**, 15–38 (2015).

20. D. A. Abanin, E. Altman, I. Bloch M. Serbyn, Colloquium: Many-body localization, thermalization, and entanglement. *Rev. Mod. Phys.* **91**, 021001 (2019).

21. F. Alet, N. Laflorencie, Many-body localization: An introduction and selected topics. *C. R. Phys.* **19**, 498–525 (2018).

22. E. Altman, R. Vosk, Universal dynamics and renormalization in many-body-localized systems. *Annu. Rev. Condens. Matter Phys.* **6**, 383–409 (2015).

23. D. Abanin, J. Bardarson, G. De Tomasi, S. Gopalakrishnan, V. Khemani, S. Parameswaran, F. Pollmann, A. Potter, M. Serbyn R. Vasseur, Distinguishing localization from chaos: Challenges in finite-size systems. *Ann. Phys.* **427**, 168415 (2021).

24. J. Zhang, P. W. Hess, A. Kyprianidis, P. Becker, A. Lee, J. Smith, G. Pagano, I.-D. Potirniche, A. C. Potter, A. Vishwanath, N. Y. Yao C. Monroe, Observation of a discrete time crystal. *Nature* **543**, 217–220 (2017).

25. A. Kyprianidis, F. Machado, W. Morong, P. Becker, K. S. Collins, D. V. Else, L. Feng, P. W. Hess, C. Nayak, G. Pagano, N. Y.Yao C. Monroe, Observation of a prethermal discrete time crystal. *Science* **372**, 1192–1196 (2021).

26. J. Rovny, R. L. Blum S. E. Barrett, Observation of discrete-time-crystal signatures in an ordered dipolar many-body system. *Phys. Rev. Lett.* **120**, 180603 (2018).

27. S. Pal, N. Nishad, T. S. Mahesh G. J. Sreejith, Temporal order in periodically driven spins in star-shaped clusters. *Phys. Rev. Lett.* **120**, 180602 (2018).

28. J. O'Sullivan, O. Lunt, C. W. Zollitsch, M. L. W. Thewalt, J. J. L. Morton A. Pal, Signatures of discrete time crystalline order in dissipative spin ensembles. *New J. Phys.* **22**, 085001 (2020).

29. J. Randall, C. E. Bradley, F. V. van der Gronden, A. Galicia, M. H. Abobeih, M. Markham, D. J. Twitchen, F. Machado, N. Y. Yao T. H. Taminiau, Observation of a many-body-localized discrete time crystal with a programmable spin-based quantum simulator. *Science* **374**, 1474–1478 (2021).

30. J. Smits, L. Liao, H. T. C. Stoof P. van der Straten, Observation of a space-time crystal in a superfluid quantum gas. *Phys. Rev. Lett.* **121**, 185301 (2018).

31. S. Autti, V. B. Eltsov G. E. Volovik, Observation of a time quasicrystal and its transition to a superfluid time crystal. *Phys. Rev. Lett.* **120**, 215301 (2018).

32. K. Giergiel, A. Kosior, P. Hannaford K. Sacha, Time crystals: Analysis of experimental conditions. *Phys. Rev. A* **98**, 013613 (2018).

33. M. Ippoliti, K. Kechedzhi, R. Moessner, S. L. Sondhi V. Khemani, Many-body physics in the NISQ era: Quantum programming a discrete time crystal. *PRX Quantum* **2**, 030346 (2021).

34. C. W. von Keyserlingk, V. Khemani S. L. Sondhi, Absolute stability and spatiotemporal long-range order in floquet systems. *Phys. Rev. B* **94**, 085112 (2016).

35. D. Abanin, W. D. Roeck, W. W. Ho F. Huveneers, A rigorous theory of many-body prethermalization for periodically driven and closed quantum systems. *Commun. Math. Phys.* **354**, 809–827 (2017).

36. D. V. Else, B. Bauer C. Nayak, Prethermal phases of matter protected by time-translation symmetry. *Phys. Rev. X* **7**, 011026 (2017).

37. H. Xu, J. Zhang, J. Han, Z. Li, G. Xue, W. Liu, Y. Jin H. Yu, Realizing discrete time crystal in an one-dimensional superconducting qubit chain. arXiv:2108.00942 (2021).

38. X. Mi, M. Ippoliti, C. Quintana, A. Greene, Z. Chen, J. Gross, F. Arute, K. Arya, J. Atalaya, R. Babbush, J. C. Bardin, J. Basso, A. Bengtsson, A. Bilmes, A. Bourassa, L. Brill, M. Broughton, B. B. Buckley, D. A. Buell, B. Burkett, N. Bushnell, B. Chiaro, R. Collins, W. Courtney, D. Debroy, S. Demura, A. R. Derk, A. Dunsworth, D. Eppens, C. Erickson, E. Farhi, A. G. Fowler, B. Foxen, C. Gidney, M. Giustina, M. P. Harrigan, S. D. Harrington, J. Hilton, A. Ho, S. Hong, T. Huang, A. Huff, W. J. Huggins, L. B. Ioffe, S. V. Isakov, J. Iveland, E. Jeffrey, Z. Jiang, C. Jones, D. Kafri, T. Khattar, S. Kim, A. Kitaev, P. V. Klimov, A. N. Korotkov, F. Kostritsa, D. Landhuis, P. Laptev, J. Lee, K. Lee, A. Locharla, E. Lucero, O. Martin, Jarrod R. Mc Clean, T. M. Court, M. M. Ewen, K. C. Miao, M. Mohseni, S. Montazeri, W. Mruczkiewicz, O. Naaman, M. Neeley, C. Neill, M. Newman, M. Y. Niu, Thomas E. O' Brien, A. Opremcak, E. Ostby, B. Pato, A. Petukhov, N. C. Rubin, D. Sank, K. J. Satzinger, V. Shvarts, Y. Su, D. Strain, M. Szalay, M. D. Trevithick, B. Villalonga, T. White, Z. Jamie Yao, P. Yeh, J. Yoo, A. Zalcman, H. Neven, S. Boixo, V. Smelyanskiy, A. Megrant, J. Kelly, Y. Chen, S. L. Sondhi, R. Moessner, K. Kechedzhi, V. Khemani, P. Roushan, Observation of time-crystalline eigenstate order on a quantum processor. *Nature* **601**, 531–536 (2022).

39. S. Endo, S. C. Benjamin Y. Li, Practical quantum error mitigation for near-future applications. *Phys. Rev. X* **8**, 031027 (2018).

40. G. White, C. Hill L. Hollenberg, Performance optimization for drift-robust fidelity improvement of two-qubit gates. *Phys. Rev. Appl.* **15**, 014023 (2021).

41. H. J. Vallury, M. A. Jones, C. D. Hill L. C. L. Hollenberg, Quantum computed moments correction to variational estimates. *Quantum* **4**, 373 (2020).

42. J. Vovrosh, K. E. Khosla, S. Greenaway, C. Self, M. Kim J. Knolle, Simple mitigation of global depolarizing errors in quantum simulations. *Phys. Rev. E* **104**, 035309 (2021).

43. G. J. Mooney, G. A. L. White, C. D. Hill L. C. L. Hollenberg, Whole-device entanglement in a 65-qubit superconducting quantum computer. *Adv. Quant. Technol.* 2100061 (2021).

44. M. Malekakhlagh, E. Magesan D. C. McKay, First-principles analysis of cross-resonance gate operation. *Phys. Rev. A* **102**, 042605 (2020).

45. L. C. G. Govia, G. J. Ribeill, D. Ristè, M. Ware H. Krovi, Bootstrapping quantum process tomography via a perturbative ansatz. *Nat. Commun.* **11**, 1084 (2020).
